# Proton-Prompted Ligand Exchange to Achieve High-Efficiency CsPbI_3_ Quantum Dot Light-Emitting Diodes

**DOI:** 10.1007/s40820-024-01321-8

**Published:** 2024-02-01

**Authors:** Yanming Li, Ming Deng, Xuanyu Zhang, Lei Qian, Chaoyu Xiang

**Affiliations:** 1 Laboratory of Advanced Nano-Optoelectronic Materials and Devices, Qianwan Institute of CNITECH, Ningbo, 315300 People’s Republic of China; 2 Division of Functional Materials and Nanodevices, Ningbo Institute of Materials Technology and Engineering, Chinese Academy of Sciences, Ningbo, 315201 People’s Republic of China; 3 Zhejiang Provincial Engineering Research Center of Energy Optoelectronic Materials and Devices, Ningbo Institute of Materials Technology & Engineering, Chinese Academy of Sciences, Ningbo, 315201 People’s Republic of China; 4 Faculty of Electrical Engineering and Computer Science, Ningbo University, Ningbo, 315211 Zhejiang People’s Republic of China; 5 University of Nottingham Ningbo China, Ningbo, 315100 People’s Republic of China

**Keywords:** CsPbI_3_ perovskite quantum dots, Light-emitting diodes, Ligand exchange, Proton-prompted in-situ exchange

## Abstract

CsPbI_3_ perovskite quantum dots (QDs) are ideal materials for the next generation of red light-emitting diodes. However, the low phase stability of CsPbI_3_ QDs and long-chain insulating capping ligands hinder the improvement of device performance. Traditional in-situ ligand replacement and ligand exchange after synthesis were often difficult to control. Here, we proposed a new ligand exchange strategy using a proton-prompted in-situ exchange of short 5-aminopentanoic acid ligands with long-chain oleic acid and oleylamine ligands to obtain stable small-size CsPbI_3_ QDs. This exchange strategy maintained the size and morphology of CsPbI_3_ QDs and improved the optical properties and the conductivity of CsPbI_3_ QDs films. As a result, high-efficiency red QD-based light-emitting diodes with an emission wavelength of 645 nm demonstrated a record maximum external quantum efficiency of 24.45% and an operational half-life of 10.79 h.

**Supplementary Information:**

The online version contains supplementary material available at 10.1007/s40820-024-01321-8.

## Highlights

- A new proton-promoted in situ ligand exchange strategy based on CsPbI_3_ quantum dots.
- The ligand exchange strategy maintains the quantum confinement effect of quantum dots and significantly improves the stability and photophysical properties of CsPbI_3_ quantum dots.
- The performance of light-emitting diodes based on CsPbI_3_ quantum dots is significantly improved, the external quantum efficiency is increased from 18.63% to 24.45%, and the half-operational lifetime is increased by 70 times.

**Supplementary Information:**

The online version contains supplementary material available at 10.1007/s40820-024-01321-8.

## Graphical abstract

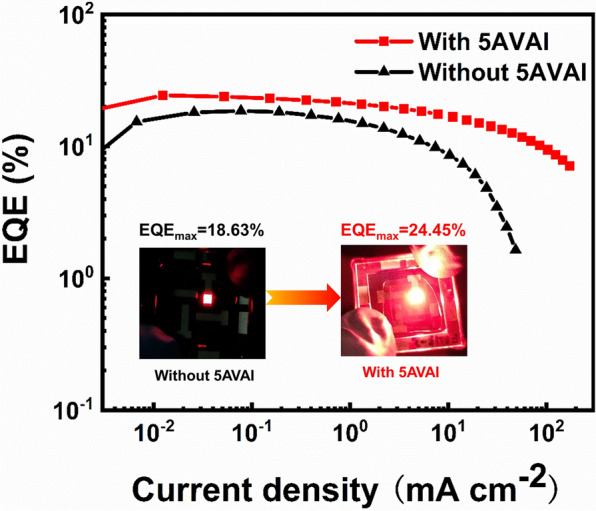

## Introduction

Metal halide perovskite nanocrystals (NCs) are considered the next-generation materials of light-emitting diodes (LEDs) due to their high fluorescence quantum yield (PLQY), continuously adjustable band gap, high color purity, and solution processing [1–5]. CsPbI_3_ perovskite NCs with excellent thermal stability and ideal band gap (− 1.7 eV) are widely used in red light-emitting diodes and solar cells (SCs) [6–8]. The size control of small CsPbI_3_ quantum dots (QDs) by quantum confinement effect makes it possible to meet the red standard for wide color gamut high-definition Rec. 2020 displays [9–11]. However, the high specific surface area of the small-size CsPbI_3_ QDs brings a high density of non-radiative recombination defects [12]. Traditionally, it is difficult for long-chain organic ligands, such as oleylamine (OA) and oleylamine (OAm), to sufficiently passivate the defects on the surface of small QDs due to the large steric hindrance. In addition, the fatty long-chain also hinders the charge transfer between QDs [13, 14]. Thus, replacing the long-chain ligands with short ligands is the key to improving the performance of LEDs devices based on small-size CsPbI_3_ QDs.

In-situ ligand replacement and ligand exchange after synthesis were two common methods to replace the long-chain insulating ligands [15–17]. However, balancing the colloid stability and short ligand passivation is a challenge while maintaining the CsPbI_3_ QDs confinement. During the synthesis reaction, the length of the fatty chain and the binding energy of the functional group of the ligands strongly affected the shape and size of QDs [18–20]. Besides, the low boiling point of short-chain ligands was hard to use in high-temperature thermal injection reactions. The ligand exchange after QDs synthesis requires polar solvents to remove the original long-chain ligands [21]. However, the high-ionic structure and low phase stability of perovskite QDs limit the selection of ligand solvents that can be used [22, 23]. Strong polar solvents, such as methanol, will damage the structural stability of perovskite nanocrystals. The loss of ligands during the post-treatment leads to agglomeration or even decomposition of CsPbI_3_ QDs [24, 25]. Therefore effective ligand exchange strategy is an important way to improve the stability and charge transfer performance of perovskite QDs, especially for small-size CsPbI_3_ QDs.

Here, we reported a new strategy of proton-prompted short-chain ligand exchange in the reaction cooling stage of perovskite QDs synthesis. Hydroiodic acid (HI) provided protons and dissolved a short-chain ligand 5-aminopentanoic acid (5AVA), which traditionally can only dissolve in strongly polar solvents. The introduction of protons triggered the desorption of long-chain oleylamine (OA) and oleylamine (OAm) ligands on the surface of QDs. At the same time, the amine functional groups of 5AVA ligands were protonated, which promoted their binding to the QDs surface. Meanwhile, iodine ions provided an iodine-rich environment and maintained the size of CsPbI_3_ QDs. Through this strategy, we synthesized stable small-size CsPbI_3_ QDs, and the ligand exchange did not affect the crystal structure and shape of QDs. Short-chain 5AVA ligands with bifunctional groups improved the optical properties of CsPbI_3_ QDs as well as the conductivity of the QD films. Based on the CsPbI_3_ QDs with 5AVA ligands, high-efficiency red QD-based light-emitting diodes (QLEDs) with a peak quantum efficiency (EQE) of 24.45% and stable electroluminescence at 645 nm emission wavelength were achieved. Compared with the control device, the brightness of the device was significantly improved to 7494 cd m^−2^. And the half-operational lifetime was improved to 10.79 h, which was 70 times higher than the control device.

## Experimental Section

### Materials

Lead iodide (PbI_2_, Macklin, 99.999%), zinc iodide (ZnI_2_, Aladdin, 99.99%), cesium carbonate (Cs_2_CO_3_, Sigma-Aldrich), 5-aminovaleric acid (5AVA, Aladdin, 97%), hydroiodic acid (HI, Aladdin, 55.0%–58.0%), octadecene (ODE, Sigma-Aldrich, 90%), oleic acid (OA, Sigma-Aldrich, 90%), oleylamine (OAm, Aladdin, 90%), n-hexane (Aladdin, 98%), n-octane (Aladdin, 99%), methyl acetate (Sinopharm Chemical Reagent Co., Ltd. (SCRC), 98%), ethyl acetate (Aladdin, 99.9%), poly(3,4-ethylenedioxythiophene) polystyrene sulfonate (PEDOT:PSS, 4083, Xi’an Polymer Light Technology Corp.), poly[bis(4-phenyl)(2,4,6-trimethylphenyl)amine](PTAA, Lingzhi Technology Co., Ltd.), 2,4,6-tris[3-(diphenylphosphinyl)phenyl]-1,3,5-triazine (PO-T2T, Xi’an Polymer Light Technology Corp.), 3,3′-[5′-[3-(3-pyridinyl)phenyl][1,1′:3′,1″-terphenyl]-3,3″-diyl]bispyridine (TmPyPB, Xi’an Polymer Light Technology Corp.), lithium fluoride (LiF, Xi’an Polymer Light Technology Corp., 99.9%), which were directly used.

### Synthesis of CsPbI_3_ QDs

#### Preparation of 5AVAI Ligand Solution

The 5AVA of (0.1, 0.2, 0.3) mmol (M) was dissolved in 1.5 times HI, and then 1 mL ethyl acetate was added to prepare the 5AVAI solution. The 5AVAI solution was heated to 80 °C for subsequent ligand treatment.

#### Preparation of Cs-OA Solution

144 mg of Cs_2_CO_3_, 11 mL of ODE, and 6 mL of OA were loaded into a 50 mL 3-neck flask. The mixture is dried for 15 min at room temperature in a vacuum. Then, the mixture was heated to 100 °C until a transparent solution was formed and heated at 100 °C for 30 min under argon flow. The obtained Cs-OA solution was stored in an argon atmosphere and preheated to 100 °C for subsequent perovskite QD synthesis.

#### ***Synthesis and Purification of CsPbI***_***3***_*** QDs Treated with 5AVAI***

170 mg PbI_2_, 345 mg ZnI_2_, and 6 mL ODE were loaded into a 50 mL three-necked flask and dried under argon flow at 120 °C for 1 h. Then, 1 mL OA and 2 mL OAm were injected at 120 °C under argon flow. The temperature was increased to 150 °C and Cs-oleate (2.2 mL of the stock solution prepared as described above) was swiftly injected. After five seconds, the reaction mixture was immediately cooled to 100 °C by immersing in a cold water bath, the prepared 5AVAI ligand solution was swiftly injected, and then the reaction mixture was cooled down to room temperature.

The crude solution was centrifuged at 5000 rpm for 1 min to remove unreacted precursor precipitate and then transferred to two 50 mL centrifuge tubes equally. Anti-solvent ethyl acetate and methyl acetate were added to the obtained crude solution (the volume ratio of QDs solution, ethyl acetate, and methyl acetate was 1:1:3), and then centrifuged at 7000 rpm for 2 min. The obtained precipitate containing CsPbI_3_ QDs was re-dispersed in 1 mL of hexane and centrifuged at 5000 rpm for 1 min to remove non-perovskite precipitates. The preliminary purified QDs solution of 1 mL was added to 6 mL methyl acetate and 6 mL ethyl acetate to precipitate again and centrifuged at 4000 rpm for 5 min. The obtained CsPbI_3_ QDs were redispersed in 1 mL of octane and centrifuged at 5000 rpm for 1 min. Finally, the QDs solution was filtered by a 0.22 μm poly(tetrafluoroethylene) filter.

#### ***Synthesis and Purification of CsPbI***_***3***_*** QDs without 5AVAI Treatment***

170 mg PbI_2_, 345 mg ZnI_2_, and 6 mL ODE were loaded into a 50 mL three-necked flask and dried under argon flow at 120 °C for 1 h. Then, 1 mL OA and 2 mL OAm were injected at 120 °C under argon flow. The temperature was increased to 150 °C and Cs-oleate (2.2 mL of the stock solution prepared as described above) was swiftly injected. After five seconds, the reaction mixture was immediately cooled down to room temperature by immersing it in a cold-water bath.

The subsequent purification process is the same as the purification of CsPbI_3_ QDs treated by 5AVAI.

These synthesized QDs samples are stored for further characterization and fabrication of LEDs devices.

### Fabrication and Characterization of CsPbI_3_ QDs-Based LEDs

The ITO-coated glass substrates were ultrasonicated with ITO cleaning solution, deionized water, acetone, isopropanol, and ethanol for 30 min respectively. Then, these substrates were put into an ultraviolet-ozone cleaner for 15 min. PEDOT:PSS solution was spin-coated on ITO-coated glass substrates at 4500 rpm for 45 s and annealed at 150 °C for 20 min in air. The coated substrate was moved into a nitrogen glove box. PTAA solution (8 mg mL^−1^) in chlorobenzenes was spin-coated on the PEDOT:PSS layer at 2000 rpm for 45 s and annealed in a nitrogen atmosphere at 120 °C for 20 min. The CsPbI_3_ QDs or 5AVAI-CsPbI_3_ QDs in octane (10 mg mL^−1^) were spin-coated onto the PTAA layer by the spin-coater at 4000 rpm for 45 s and annealed at 60 °C for 5 min. Finally, under the condition of high vacuum (−2 × 10^−4^ Pa), a 6 nm TmPyPB layer, 40 nm PO-T2T layer, 1 nm LiF layer, and 100 nm Al electrode were deposited by thermal evaporation system. The device's active area was 4 mm^2^. Subsequently, the performance of the fabricated LED device is tested. The EL spectrum was collected by Ocean Optics USB 2000 + spectrometer, and the *J-L-V* characteristic of the device was measured by Keithley 2400 source table. The life of LEDs devices was measured by using a life test system (Guangzhou Jinghe Equipment Co., Ltd.) under ambient conditions.

### Characterizations

The ultraviolet–visible (UV–vis) absorption spectrum of the QD solution was obtained by the PerkinElmer instrument. Photoluminescence (PL) spectrum of QD solution was measured on HORIBA fluorescence spectrometer (FL3-111) and excited at 365 nm. PLQY of QD solution was measured by Otsuka QE2100 device, diluted with hexane in quartz cuvette, and excited at 365 nm. Transmission electron microscope (TEM) images were obtained by Talos F200X instrument, and the accelerating voltage was 200 kV. Atomic force microscope (AFM) images of QD thin films were collected in tapping mode with a Dimension ICON instrument. Powder X-ray diffraction (XRD) patterns were recorded using a D8 ADVANCE diffractometer with Cu Kα radiation (λ = 1.54178 Å). Fourier transform infrared (FTIR) spectrum of QDs powder was obtained by Fourier infrared spectrometer (IS50). The time-resolved photoluminescence luminescence decay spectra of QDs were obtained by FL3-111 spectrometer.

## Results and Discussion

### Proton-Prompted Ligand Exchange

**Fig. 1:**
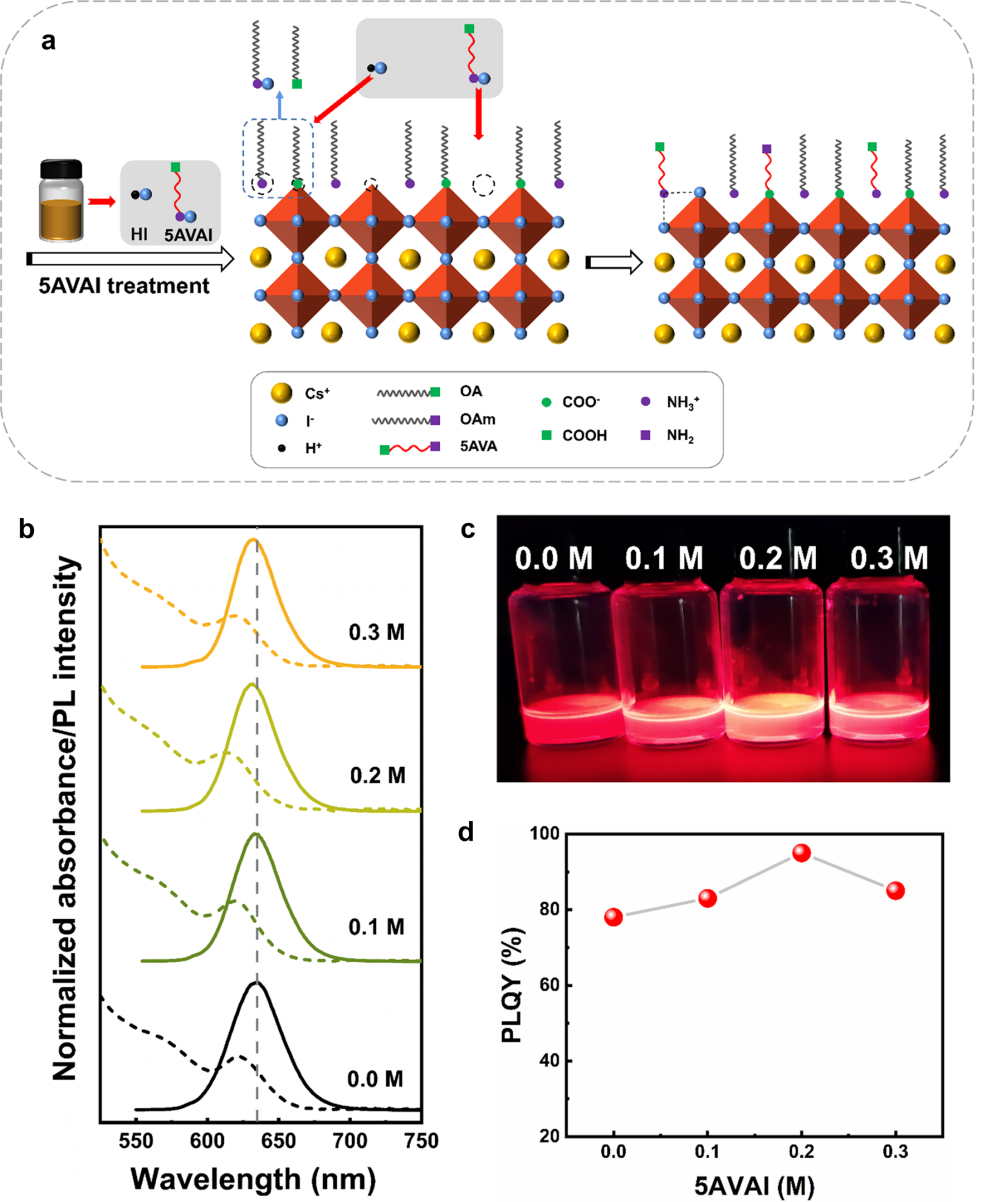
**a** Strategy diagram of in situ exchange between 5AVA ligand and OA/OAm ligand driven by HI. **b** The UV–vis absorption and fluorescence spectra of CsPbI_3_ QDs purified once with different 5AVAI content. **c** Photos of CsPbI_3_ QDs treated with different 5AVAI amounts under ultraviolet light (365 nm). **d** PLQY of CsPbI_3_ QDs treated with different amounts of 5AVAI

CsPbI_3_ QDs with or without 5AVA were synthesized by a modified thermal injection method (see the experimental part for details). 5AVA ligands were dissolved in excess HI solution to form 5-Ammonium valeric acid iodide (5AVAI) as the precursor (Fig. S1a). Bright CsPbI_3_ QDs were synthesized by injection 5AVAI at the cooling stage after the nucleation of CsPbI_3_ QDs (Fig. S1b). There is a highly dynamic binding between the surface-capped ligands of perovskite QDs [26]. For the traditional OA and OAm capped QDs, there is a balance between the ionization form and molecular form of these ligands (OA^−^  + OAmH^+^  ⇌ OAH + OAm or OAmH^+^  + I^−^  ⇌ OAm + HI) [27, 28]. This dynamic binding reaction leads to the desorption of ligands during the separation and purification of QDs, resulting in a decrease in the stability and optical properties of QDs. In the proton-prompted strategy, the protons provided by excess HI drive the dynamic equilibrium transfer of OA^−^ and OAmH^+^ ligands bound to QDs surface to molecular form. Then 5AVAI ligands with bifunctional groups bind to surface sites left by OA and OAm ligands. The CsPbI_3_ QDs synthesized by HI treatment (without 5AVA) almost decomposes (Fig. S1c), which is attributed to the proton provided by HI which promotes the shedding of OA and OAm ligands on the surface of QDs. In addition, the I^−^ from HI can fill the I^−^ vacancy defects on the surface of QDs. Figure 1a illustrates the strategy of in situ exchange of the HI-driven 5AVA ligands with the OA/OAm ligands.

The optical properties of CsPbI_3_ QDs were characterized to study the influence of 5AVAI ligands. As the amount of 5AVAI increased, the first exciton absorption peak and PL peak shifted to a shorter wavelength, as shown in Fig. 1b. The blue shift of the absorption peak and PL peak is attributed to the decrease in QDs size due to the increase of iodine ions in the reaction system [29]. The photos of CsPbI_3_ QDs synthesized with different 5AVAI amounts under ultraviolet light (365 nm) are shown in Fig. 1c. With the increase of the 5AVAI amount, the PLQY of QDs increased, reaching the maximum value of 95% at 0.2 M (Fig. 1d). These results show that the introduction of 5AVAI in the synthesis process can reduce the size of CsPbI_3_ QDs, effectively passivate the surface defects of CsPbI_3_ QDs, and improve the optical properties of QDs. However, for the synthesized CsPbI_3_ QDs, the CsPbI_3_ QDs are decomposed after introducing 5AVAI ligands for ligand exchange (Fig. S2), which is attributed to the strong polarity of HI leading to the shedding of ligands on the surface of the QDs.

**Fig. 2:**
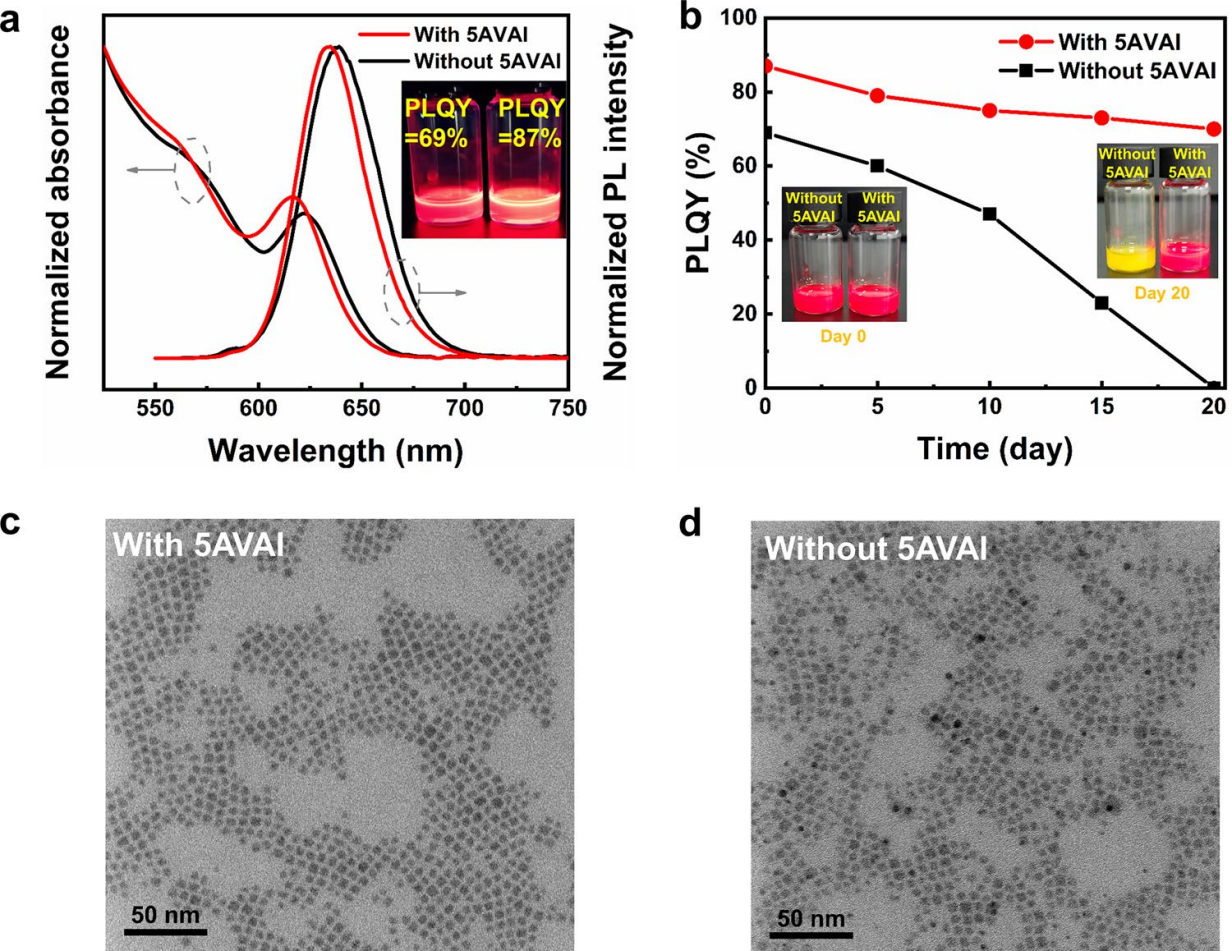
**a** The UV–vis absorption and fluorescence spectra of CsPbI_3_ QDs with and without 5AVAI were purified twice. The inset images show the photo of QDs (left: without 5AVAI; right: with 5AVAI) under ultraviolet (365 nm) and the corresponding PLQY value. **b** Stability of CsPbI_3_ QD with and without 5AVAI under ambient conditions (temperature of 25 ± 5 °C and humidity of 50 ± 10%), the inset images show the photos of two kinds of QDs after different storage days. TEM images of CsPbI_3_ QDs **c** with and **d** without 5AVAI

The optical properties and morphology of CsPbI_3_ QDs synthesized with 0.2 M 5AVAI and without 5AVAI were compared. With the same synthesis condition, the PL peak wavelength with 5AVAI is shorter than that of PL without 5AVAI treatment. The full width at half maximum (FWHM) is narrower (38 and 42 nm for with 5AVAI and without 5AVAI, respectively), which indicates that QDs treated by 5AVAI has smaller size and more uniform size distribution. Iodine-rich environment inhibits the growth and size distribution broadening of QDs caused by Ostwald ripening. The PLQY of QDs with 5AVAI is 87%, while the PLQY of QDs without 5AVAI treatment is 69%, indicating that 5AVAI effectively passivates the surface defects of QDs. Furthermore, we studied the stability of QDs under ambient conditions (Fig. 2b). The QDs treated by 5AVAI were still more than 70% after 20 days, while the QDs without 5AVAI treatment decomposed after 20 days. These results show that 5AVAI ligands exchange the dynamical OA and OAm ligands on the QDs surface, which effectively passivate the surface defects of QDs and improve the stability of QDs. The effect of 5AVAI on the morphology of QDs was characterized by a TEM. Figure 2c, d shows the TEM images of CsPbI_3_ QDs with and without 5AVAI, the 5AVAI-treated QDs maintain cubic shape and good monodispersity. In addition, compared with QDs without 5AVAI treatment (5.88 ± 1.16 nm), 5AVAI treatment has a smaller size (5.29 ± 0.76 nm) and a more uniform size distribution (the particle size distribution of QDs is shown in Fig. S3).

### Interaction Between 5AVAI and CsPbI_3_ QDs

**Fig. 3:**
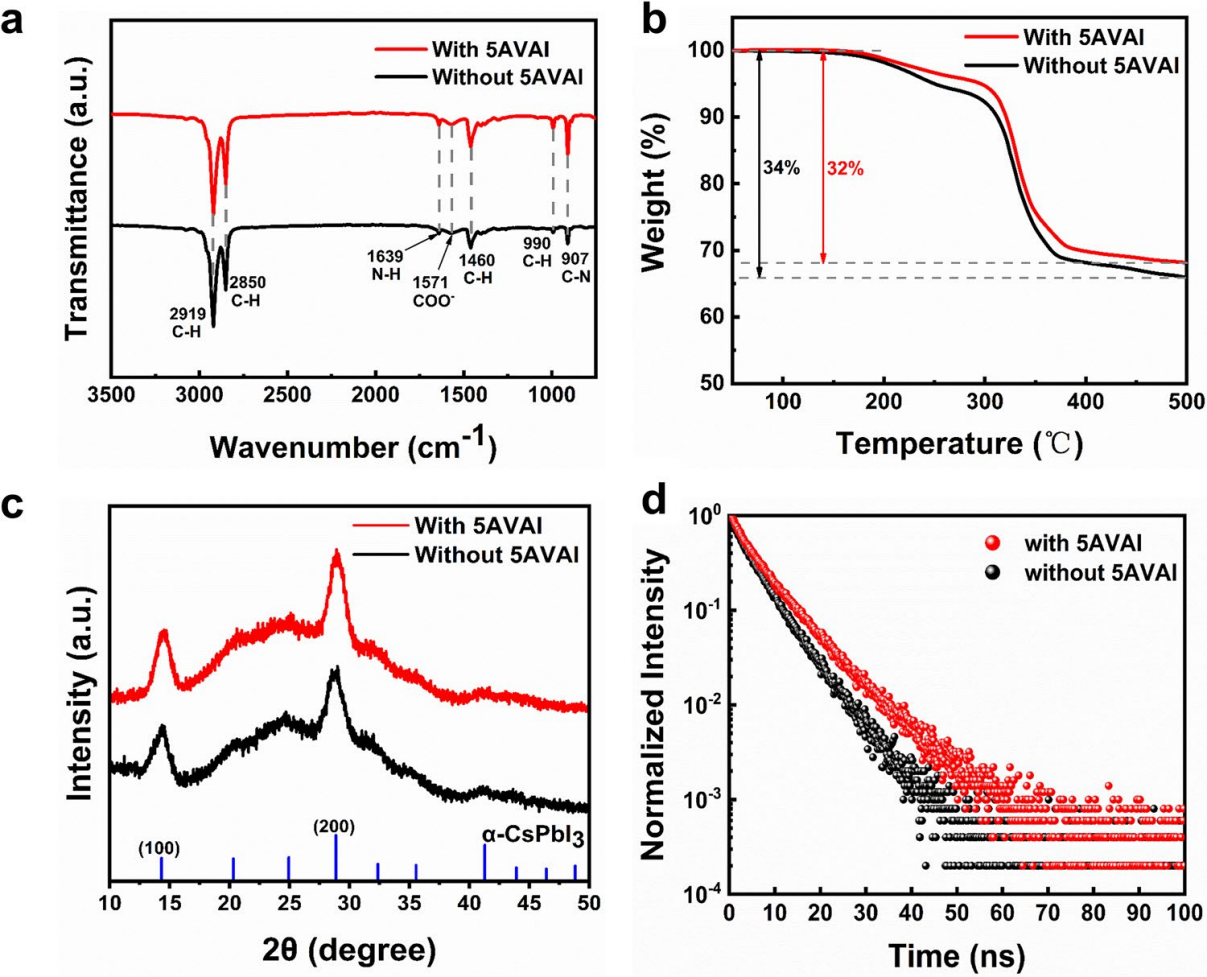
**a** FTIR spectra of CsPbI_3_ QDs with and without 5AVAI. **b** TGA of CsPbI_3_ QDs with and without 5AVAI. **c** XRD spectra of CsPbI_3_ QDs films with and without 5AVAI. **d** Time-resolved photoluminescence (TRPL) decay of CsPbI_3_ QDs films with and without 5AVAI

To determine the interaction between QDs and 5AVAI ligands, the ligands on the surface of QDs were characterized. As shown in Fig. 3a, the signal peak at 1639 cm^−1^ represents the N–H bending vibration of the amine functional group [30], and the peak at 1571 cm^−1^ is due to the asymmetric stretching vibration peak of carboxylate (COO^−^) [31], which indicates that there are OA, OAm or 5AVAI ligands on QDs surface. After 5AVAI ligand treatment, the peak of the C–N stretching vibration signal at 907 cm^−1^ is enhanced [32, 33], indicating that the ligand containing amino functional groups on the QDs surface was increased due to the combination of 5AVAI ligand and QDs. In addition, the high-resolution XPS spectrum (Fig. S4) shows that the binding energy of Pb 4*f* in CsPbI_3_ QDs treated with 5AVAI is significantly increased compared with the QDs without 5AVAI treatment. This shows that 5AVAI has a strong interaction with uncoordinated lead atoms on the surface of QDs, which makes the chemical environment of QDs change significantly. TGA showed that the weight of CsPbI_3_ QDs with 5AVAI decreased by 32% at 500 °C, while the weight of CsPbI_3_ QDs without 5AVAI decreased by 34%, indicating the decrease of long-chain ligands (Fig. 3b). XRD patterns (Fig. 3c) confirmed that the crystal structure of QDs did not change after treatment with 5AVAI ligands, all were the same cubic crystal structure. Time-resolved PL decay spectrum (Fig. 3d) shows that the average fluorescence lifetimes of CsPbI_3_ QDs with and without 5VAI are 7.1 and 5.5 ns, respectively, which proves that the defects of CsPbI_3_ QDs can be effectively reduced by 5VAI ligand treatment, leading to enhanced radiation recombination. AFM was used to characterize the surface morphology of CsPbI_3_ QDs films with and without 5AVAI treatment (Fig. S5). The root mean square (RMS) roughness of CsPbI_3_ QDs film with 5AVAI is 3.25 nm, while that of CsPbI_3_ QDs film without 5AVAI is 5.73 nm. This is attributed to the exchange of some long-chain OA and OAm ligands between 5AVAI ligands, and the closer accumulation of QDs reduces the surface roughness of the films. This ensures good contact between the CsPbI_3_ QDs layer and the hole transport layer.

### Device Performance

**Fig. 4:**
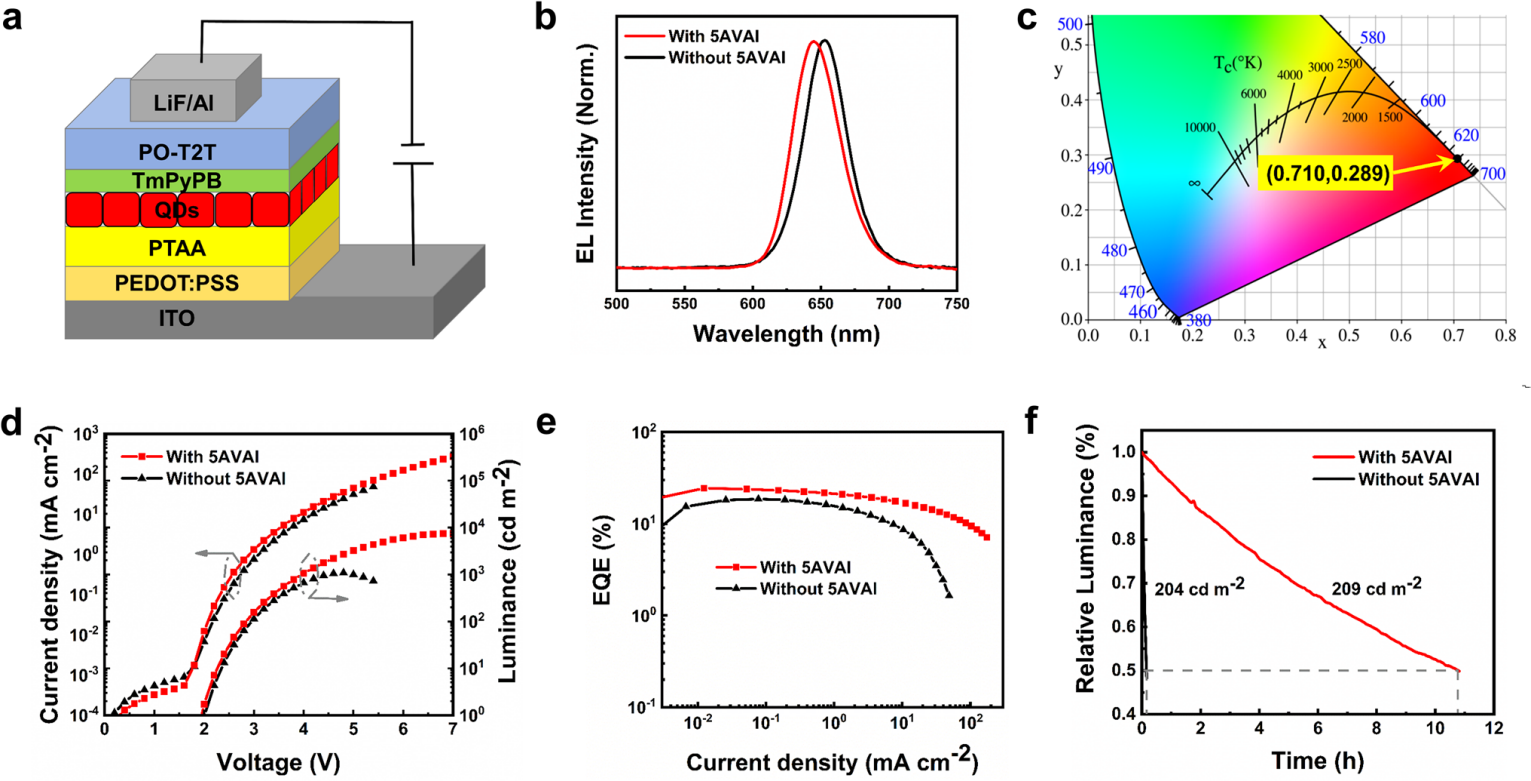
**a** QLEDs device structure diagram. **b** Normalized electroluminescence spectra of CsPbI_3_ QDs devices with and without 5AVAI treatment. **c** CIE coordinates correspond to the electroluminescence spectra of devices based on 5AVAI-treated CsPbI_3_ QDs. QLEDs operating characteristics based on CsPbI_3_ QDs with and without 5AVAI: **d** current density and luminance-voltage curve; **e** EQE-current density curve. **f** QLEDs stability based on CsPbI_3_ QDs with and without 5AVAI treatment

QLEDs devices using CsPbI_3_ QDs films with and without 5AVAI treatment as emitting layers were fabricated. Figure 4a illustrates the QLEDs device structure which is composed of ITO/PEDOT:PSS/PTAA/CsPbI_3_ QDs/TmPyPB/PO-T2T/LiF/Al, where ITO is the anode and PEDOT:PSS is the hole injection layer, PTAA is the hole transport/electron barrier layer, TmPyPB/PO-T2T is the electron transport layer, and LiF/Al is the cathode. The thin TmPyPB electron transport layer inhibits the quenching of excitons produced in the QDs emission layer by the POT2T layer (Fig. S6). The cross-sectional TEM image of the device is shown in Fig. S7. According to the Tauc curve (Fig. S8) and ultraviolet photoelectron spectroscopy (UPS) diagram (Fig. S9) of the thin film of CsPbI_3_ QDs with and without 5AVAI treatment, the device energy level diagrams of all functional layers are obtained (Fig. S10). Figure 4b shows the normalized electroluminescence spectra of CsPbI_3_ QDs films with and without 5AVAI ligand treatment, with electroluminescence peaks at 645 and 651 nm, respectively. The corresponding CIE 1931 color coordinate of CsPbI_3_ QDs treated by 5AVAI ligand is (0.710, 0.289) (Fig. 4c). In the range of applied bias voltage (3.1–5.8 V), the EL spectrum of QLEDs based on CsPbI_3_ QDs treated with 5VAI showed excellent color stability, while the EL spectrum of devices without 5VAI treatment shifted slightly at high voltage (Fig. S11).

Figure 4d shows the current density-voltage-brightness (*J-V-L*) characteristics of champion QLEDs based on these two kinds of CsPbI_3_ QDs. The high-quality thin film of QDs treated by 5AVAI makes the device have lower leakage current density. QLEDs based on 5AVAI treatment show significantly higher current density and brightness, and the maximum luminance is increased from 1090 to 7494 cd m^−2^. This is attributed to the exchange of short-chain 5AVAI ligands between long-chain OA and OAm ligands, which increases the charge transfer between CsPbI_3_ QDs and improves the performance of the device. The measurement of space-charge-restricted-current (SCLC) (Fig. S12) shows that the charge mobility of QDs films after 5AVAI treatment is increased. The peak EQE of QLEDs based on CsPbI_3_ QD with and without 5AVAI is 24.45% and 18.63% (Fig. 4e), respectively, and the devices treated with 5AVAI show lower efficiency rolling at high current density. The improvement in device performance is attributed to that 5AVAI passivates the surface defects of QDs and inhibits non-radiative recombination. In addition, the short-chain 5AVAI ligand improves the conductivity of QDs thin films and balances the carrier transport. Figure S6 shows the EQE histogram of 20 devices, which shows good repeatability. Finally, we evaluate the operational stability of QLEDs (Fig. 4f). At the initial brightness of 209 cd m^−2^, the T_50_ (the time required to decay to 50% of its initial value) of the CsPbI_3_ QDs device with 5AVAI treatment is about 10.79 h, and that of the QDs device without 5AVAI treatment is 0.15 h (the initial brightness is 204 cd m^−2^). After 5AVAI ligand treatment, the T_50_ of the device is improved by about 70 times, which improves the operation stability of the device. The performance of our LEDs devices represents the best performance of bright red (EL < 650 nm) PeLEDs (Table S1).

## Conclusions

In summary, we have developed a proton-prompted ligand exchange strategy on the surface of CsPbI_3_ QDs, and the effective ligand exchange significantly improved the performance of the device. In this strategy, HI provides proton-driven OA and OAm ligands desorption dynamically bound to QDs surface and realized in-situ exchange with 5AVAI ligands. The treatment of 5AVAI ligands significantly improved the stability and optical properties of CsPbI_3_ QDs. We used these stable 5AVAI-treated CsPbI_3_ QDs to fabricate bright red QLED with narrow electroluminescence at 645 nm. Short-chain 5AVAI ligand treatment significantly increased the current density and brightness of the device, achieving a record EQE of 24.45% and the T_50_ of 10.7 h, an increase of about 70 times. Our work provides a new method for ligand exchange of small-size CsPbI_3_ QDs, and it is also an effective way to improve the performance of perovskite QLEDs.

## Supplementary Information

Below is the link to the electronic supplementary material.

Supplementary file1 (PDF 1053 KB)
