# Supplementary material for: Proton-Prompted Ligand Exchange to Achieve High-Efficiency CsPbI3 Quantum Dot Light-Emitting Diodes
Source: Nanomicro Lett. 2024 Feb 1;16:105. doi: 10.1007/s40820-024-01321-8 (PMC10834927; doi:10.1007/s40820-024-01321-8)
Supplement: Supplementary file 1 — Supplementary file1 (PDF 1053 KB) [file 40820_2024_1321_MOESM1_ESM.pdf]

Supporting Information for

Proton-Prompted Ligand Exchange to Achieve High-Efficiency $CsPbI_3$ Quantum Dot Light-Emitting Diodes

Yanming Li[1, 2,†], Ming Deng[1, 2, 4,†], Xuanyu Zhang[1, 2, 5], Lei Qian[1, 2, 3], and Chaoyu Xiang[1, 2, 3, *]

[1] Laboratory of Advanced Nano-Optoelectronic Materials and Devices, Qianwan Institute of CNITECH, Ningbo 315300, P. R. China

[2] Division of Functional Materials and Nanodevices, Ningbo Institute of Materials Technology and Engineering, Chinese Academy of Sciences, Ningbo 315201, P. R. China

[3] Zhejiang Provincial Engineering Research Center of Energy Optoelectronic Materials and Devices, Ningbo Institute of Materials Technology & Engineering, Chinese Academy of Sciences, Ningbo 315201, P. R. China

[4] Faculty of Electrical Engineering and Computer Science, Ningbo University, Ningbo, Zhejiang 315211, P. R. China

[5] University of Nottingham Ningbo China, Ningbo 315100, P. R. China

†Yanming Li and Ming Deng contributed equally to this work.

*Corresponding author. E-mail: xiangchaoyu@nimte.ac.cn (Chaoyu Xiang)

Supplementary Figures and Tables

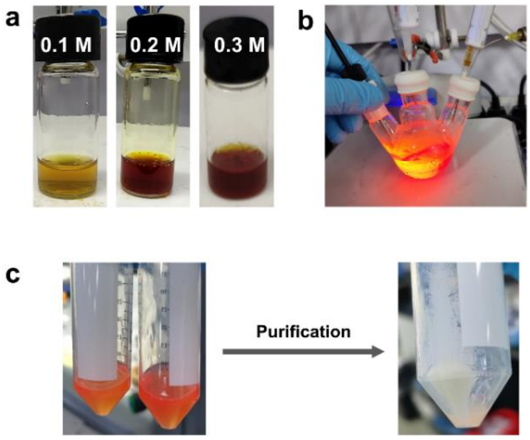

Fig. S1 a Different amounts of 5AVA (0.1, 0.2, and 0.3 M) were dissolved in excess HI and mixed with 1 ml ethyl acetate to form a 5AVAI ligand solution. **b** The photo of $CsPbI_3$ QDs synthesized by 0.2 M 5AVAI under ultraviolet light (365 nm). **c** The synthesized $CsPbI_3$ QDs were treated with HI (without 5AVA), and the QDs were purified once

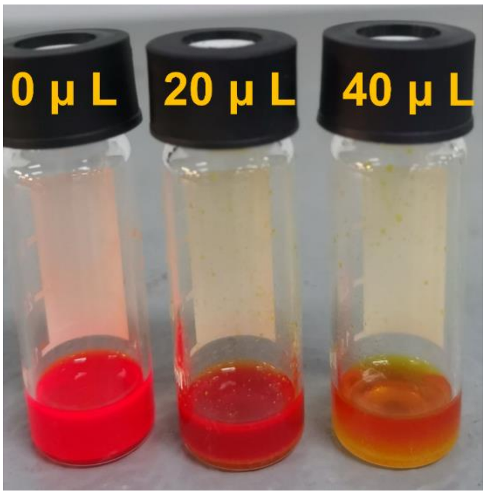

Fig. S2 $CsPbI_3$ QDs without 5AVAI treatment were added with 0, 20, and 40 μL 5AVAI ligands for ligand exchange, and the QDs were decomposed to different degrees after adding 5AVAI

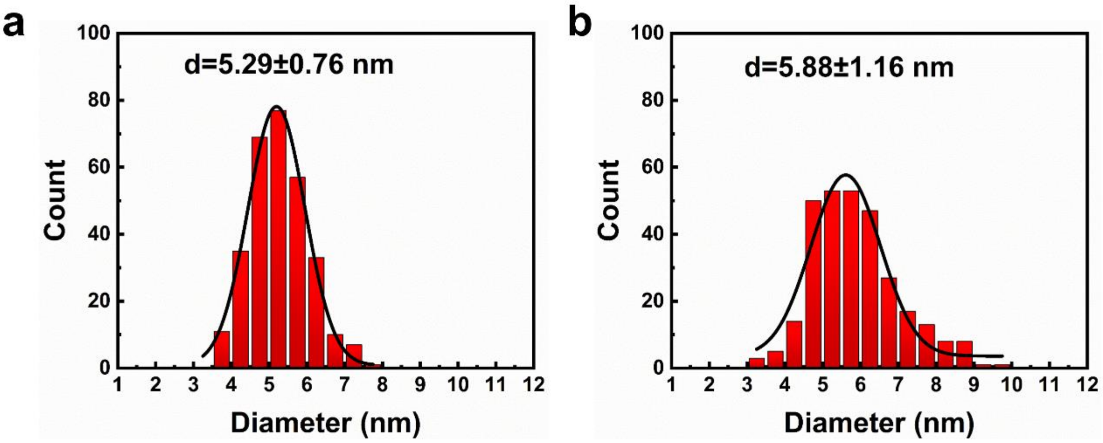

Fig. S3 The size distribution histograms of QDs corresponding to TEM images are **a** $CsPbI_3$ QDs with 5AVAI and **b** $CsPbI_3$ QDs without 5AVAI respectively

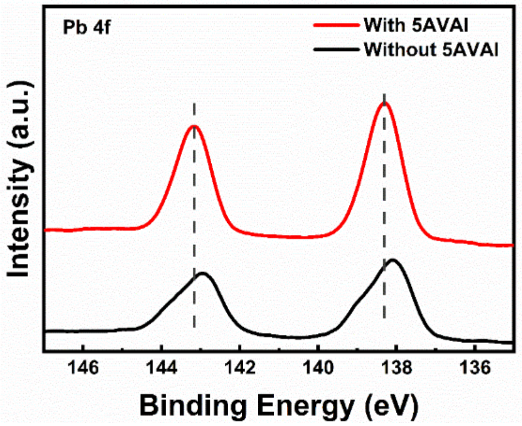

Fig. S4 X-ray photoelectron spectroscopy (XPS) was used to characterize the $CsPbI_3$ QDs with and without 5AVAI treatment, showing the high-resolution XPS spectrum of Pb 4f

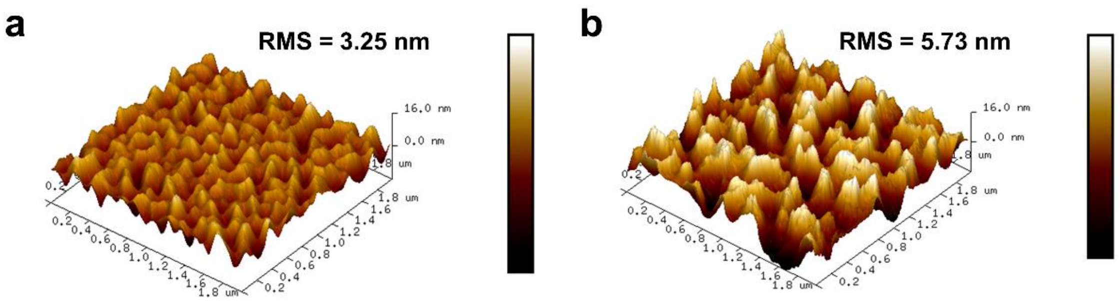

Fig. S5 AFM images of $CsPbI_3$ QDs films **a** with and **b** without 5AVAI

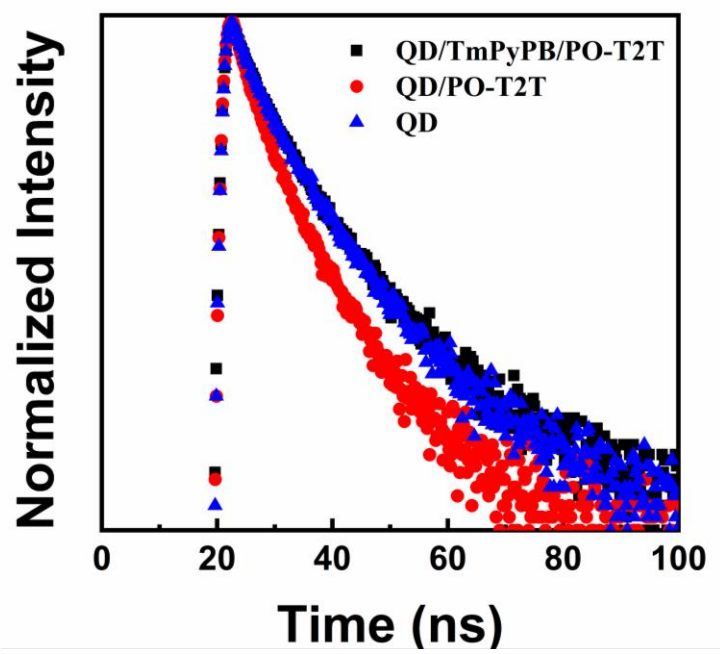

Fig. S6 Time-resolved PL delay curves of glass/QDs, glass/QDs/40 nm PO-T2T, and glass/QDs/6 nm TmPyPB/40 nm PO-T2T measured under the same conditions

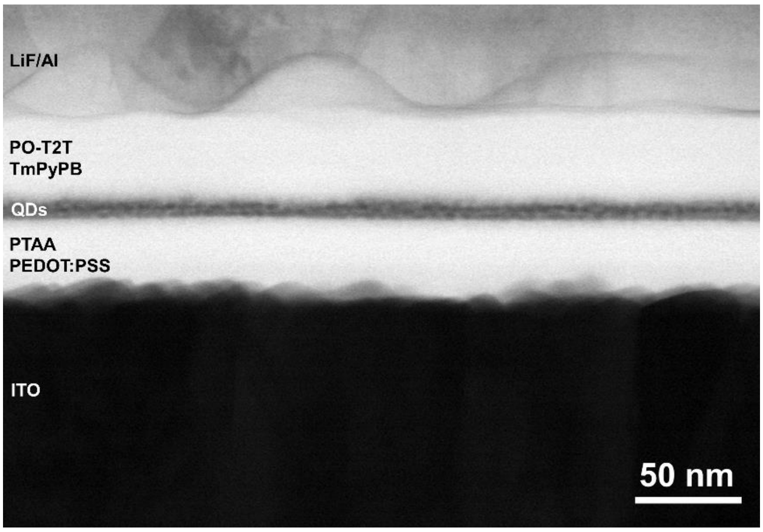

Fig. S7 Cross-sectional TEM image of LEDs based on 5AVAI-treated $CsPbI_3$ QDs

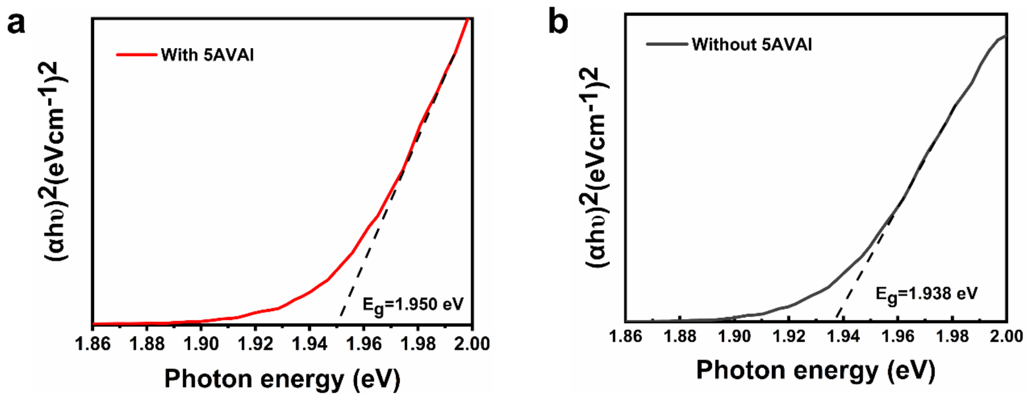

Fig. S8 Tauc plots of $CsPbI_3$ QDs **a** with and **b** without 5AVAI

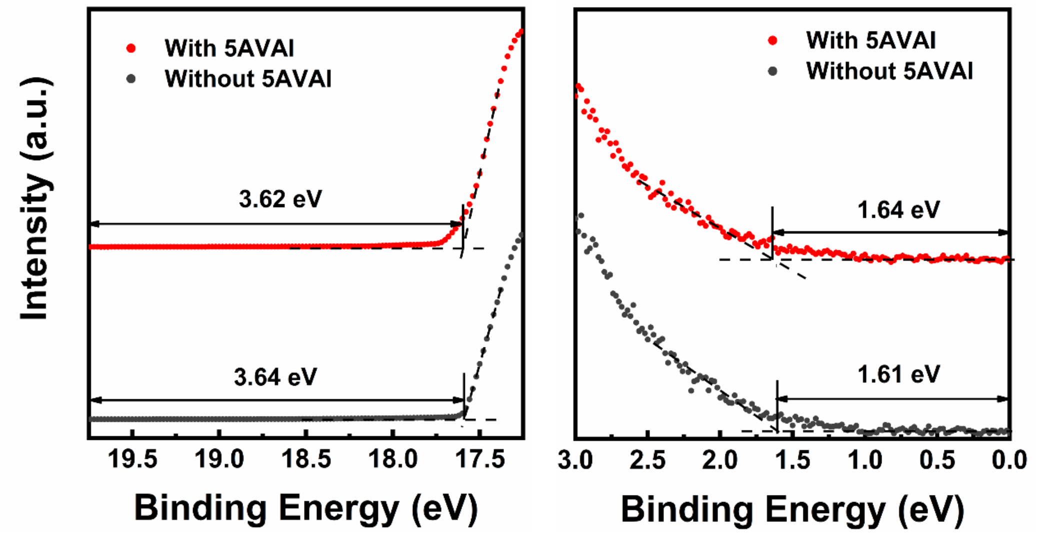

Fig. S9 UPS spectra of $CsPbI_3$ QDs with and without 5AVAI

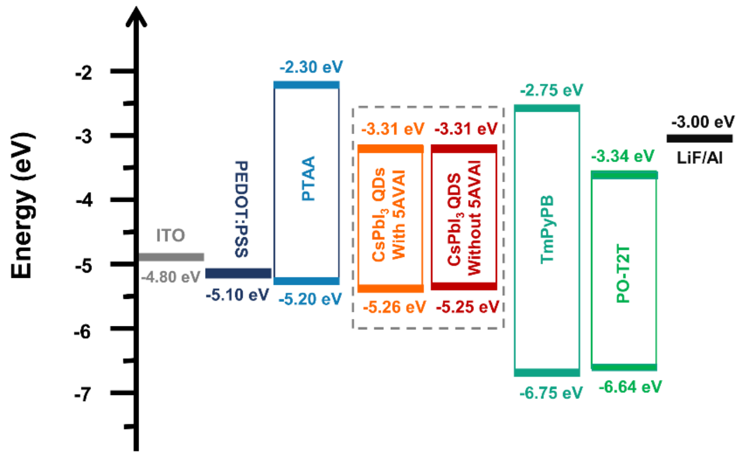

Fig. S10 Device energy level diagrams for all functional layers

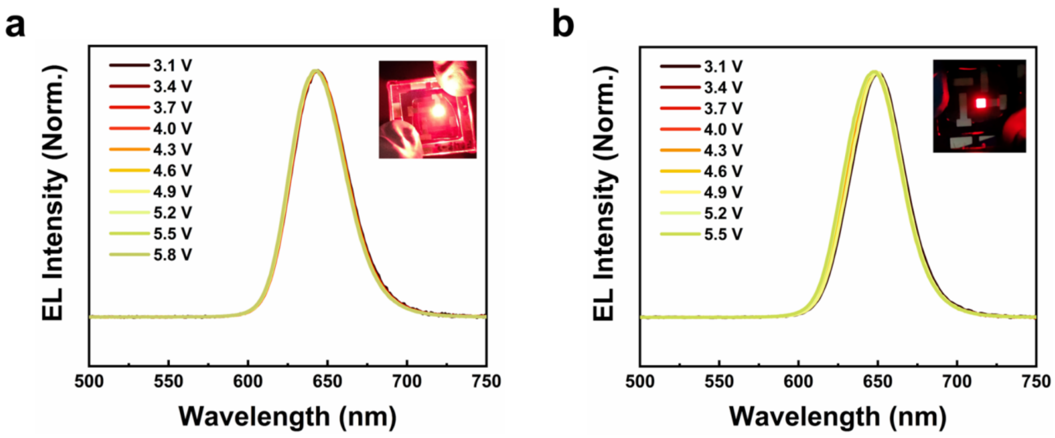

Fig. S11 Normalized EL spectra of LEDs based on **a** $CsPbI_3$ QDs treated by 5AVAI and **b** $CsPbI_3$ QDs under different driving voltages. The inset image shows the luminescence of the device at the highest driving voltage

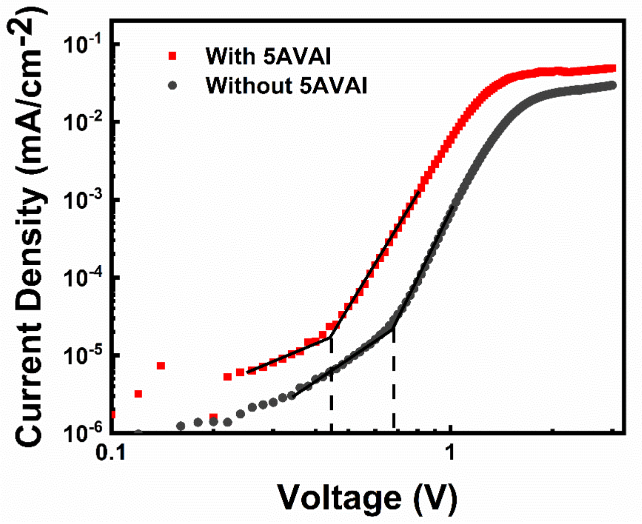

Fig. S12 J–V characteristics of hole-only devices (ITO/PEDOT:PSS/QDs/MoO_x/Al)

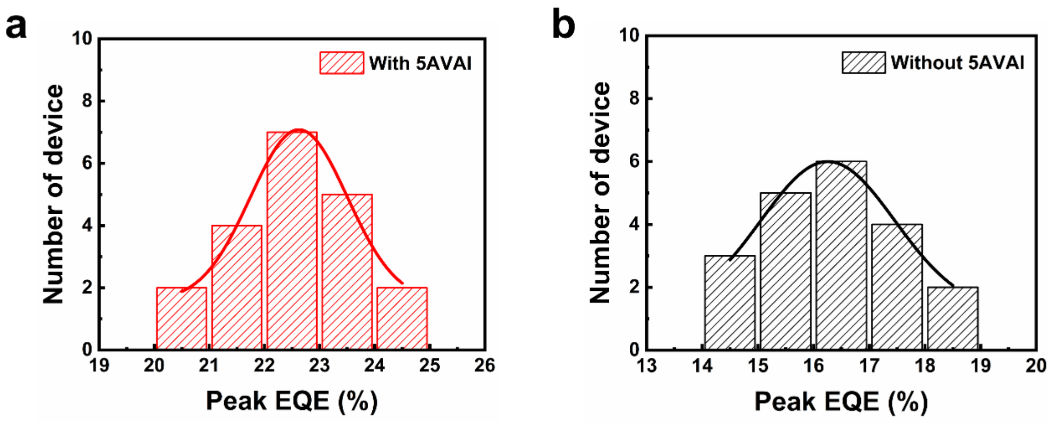

Fig. S13 LED's EQE histogram is based on $CsPbI_3$ QDs **a** with 5AVAI and **b** without 5AVAI

Table S1 Performance summary of high-performance red (EL < 650 nm) QLEDs devices based on perovskite colloidal quantum dots

**Perovskite**	**EL (nm)**	**Max. EQE (%)**	**L_{max} (cd m^{-2})**	**T_{50} (min)**	**References**
$CsPb(Br/I)_3$ QDs	630	5.1	~2200	~2 (100 cd m^{-2})	Nano Energy **62**, 434–441 (2019)
$CsPbI_3$ QDs	630	6.4	1212	78 (~30 cd m^{-2})	Nano Lett. **21**, 8756–8763 (2021)
$CsPbI_3$ QDs	634	7.1	1391	33 (200 cd m^{-2})	J. Am. Chem. Soc. **144**, 13302–13310 (2022)
$CsPb(Br/I)_3$ QDs	625	12.9	3382	-	Small **16**, 2001062 (2020)
$CsPb(Br/I)_3$ QDs	645	14.1	794	180 (100 cd m^{-2})	Nat. Photonics **12**, 681–687 (2018)
$CsPb(Br/I)_3$ QDs	646	18.2	1206	10 (100 cd m^{-2})	Adv. Funct. Mater. **31**, 2106871 (2021)
$MAPb(Br/I)_3$ QDs	620	20.3	627	342 (100 cd m^{-2})	Nature **591**, 72–77 (2021)
$CsPbI_3$ QDs	636	20.8	3775	7.4 (110 cd m^{-2})	Nano Lett. **22**, 8266–8273, (2022)
$CsPb(Br/I)_3$ QDs	637	21.8	6491	15 (120 cd m^{-2})	Adv. Funct. Mater. **33**, 2300116 (2023)
$CsPbI_3$ QDs	640	23.0	~1000	600 (200 cd m^{-2})	Angew. Chem. Int. Ed. **60**, 16164–16170 (2021)
$CsPb(Br/I)_3$ QDs	640	23.5	1510	97 (100 cd m^{-2})	Adv. Mater. **35**, 2209002 (2023)
$CsPbI_3$ QDs	645	24.45	7494	647 (209 cd m^{-2})	This work
